# Supplementary material for: Short-Term Impacts of Ambient Air Pollution on Health-Related Quality of Life: A Korea Health Panel Survey Study
Source: Int J Environ Res Public Health. 2020 Dec 7;17(23):9128. doi: 10.3390/ijerph17239128 (PMC7730868; doi:10.3390/ijerph17239128)
Supplement: Supplementary file 1 [file ijerph-17-09128-s001.pdf]

Table S1. Model selection using the cAIC to assess factors contributing to EQ-VAS scores

Model	Covariates	cAIC
Model 1	β_0+β_1(sex)+β_2(age)+β_3(education)+β_4(income)+β_5(marital status) +β_6(smoking)+β_7(alcohol consumption)	197837.9
Model 2	β_0+β_1(sex)+β_2(age)+β_3(education)+β_4(income)+β_5(marital status) +β_6(smoking)+β_7(alcohol consumption)+β_8(economic status)+β_9(chronic disease)	197675.2
Model 3 (main)	β_0+β_1(sex)+β_2(age)+β_3(education)+β_4(income)+β_5(marital status) +β_6(smoking)+β_7(alcohol consumption)+β_8(economic status)+β_9(chronic disease)+β_{10}(sleeping time)	197610.3

cAIC, conditional Akaike's information criterion.
